# Case report: A Newly Identified G-protein Subunit α-11 Loss-of-Function Variant Causing Familial Hypocalciuric Hypercalcemia Type 2 in an Italian Kindred

**DOI:** 10.3389/fendo.2026.1876004

**Published:** 2026-09-10

**Authors:** Filomena Cetani, Fabrizia Citro, Simona Borsari, Laura Pierotti, Simone Della Valentina, Chiara Sardella, Anna Dal Lago, Angela Michelucci, Maria Adelaide Caligo, Elena Pardi

**Affiliations:** 1 Department of Clinical and Experimental Medicine, University of Pisa, Pisa, Italy; 2 Endocrine Unit, University Hospital of Pisa, Pisa, Italy; 3 Laboratory of Molecular Genetics, University Hospital of Pisa, Pisa, Italy

**Keywords:** calcium/phosphate metabolism, case report, FHH2, GNA11, hypercalcemia, parathyroid-related disorders

## Abstract

**Background:**

FHH2 is the rarest subtype of familial hypocalciuric hypercalcemia disorders affecting the CaSR signaling pathway. It is caused by heterozygous loss-of-function mutations in the *GNA11* gene, encoding Gα11 protein. Only seven *GNA11* pathogenic variants have been reported so far.

**Case description:**

We report the case of a 39-year-old man referred to our outpatient clinic for a non-toxic multinodular goiter. During biochemical examination, mild hypercalcemia with normal PTH levels and low urinary calcium excretion (calcium–creatinine clearance ratio< 0.01) was detected. He had no evidence of kidney stones or nephrocalcinosis and demonstrated normal bone mineral density at dual-energy X-ray absorptiometry. A similar biochemical profile in his mother suggested FHH. Following total thyroidectomy for the goiter, the patient’s calcium and PTH levels normalized. Genetic testing identified a novel heterozygous germline frameshift variant in *GNA11* (c.980_981del, p.His327LeufsTer150) in the proband, his mother, and his 15-month-old normocalcemic daughter. This frameshift variant, which affects the critical GTPase domain, results in a pathogenic elongated protein with 116 additional amino acids, significantly altering its structure.

**Conclusions:**

In this report, we describe the eighth FHH2 kindred and highlight the biochemical heterogeneity of this rare condition. Our findings demonstrate that the normalization of serum calcium during follow-up does not necessarily exclude the diagnosis. Furthermore, we underscore the clinical importance of integrating genetic findings with longitudinal biochemical data.

## Introduction

The calcium-sensing receptor (CaSR) is a G-protein–coupled receptor that plays a central role in calcium homeostasis (1). Activation of CaSR by elevated extracellular calcium levels triggers Gα11-dependent stimulation of phospholipase C (PLC), leading to the production of inositol 1,4,5-trisphosphate (IP_3_) and subsequent release of calcium from intracellular stores. This intracellular calcium increase suppresses parathyroid hormone (PTH) secretion and reduces renal tubular calcium reabsorption.

CaSR cell surface expression is regulated by a clathrin-mediated endocytosis process, involving β-arrestin and adaptor-related protein complex 2 (AP2) (2). Disruptions in this signaling pathway elevate the calcium “set-point”—the extracellular calcium concentration required to suppress PTH secretion and promote renal calcium excretion, ultimately resulting in the familial hypocalciuric hypercalcemia (FHH) phenotype (3). FHH is a highly penetrant autosomal dominant disorder, characterized by lifelong non-progressive mild-to-moderate elevation of serum calcium levels. This condition is typically associated with normal (approximately 80%) or slightly elevated (approximately 20%) PTH levels (1, 2).

FHH is classified into three subtypes based on the specific gene mutation affecting the CaSR signaling pathway: i) FHH type 1 (FHH1) caused by loss-of-function variants in *CASR* gene; ii) FHH type 2 (FHH2) caused by mutations in *GNA11* (encoding Gα11); iii) FHH type 3 (FHH3) caused by mutations in *AP2S1* (encoding the σ subunit of AP2). FHH2 is the rarest subtype, accounting for fewer than 1% of all FHH cases. The first *GNA11* loss-of-function mutations associated with FHH2 were identified in 2013, including an in-frame deletion (p.Ile200del) and a missense mutation (p.Leu135Gln) (4). Since then, only five additional *GNA11* variants have been reported (5–9). The only validated FHH2 mouse model capable of testing targeted therapies was established by Howles and colleagues (10). They performed a genotype-driven screen on a DNA repository of over 10,000 mice mutagenized with an alkylating agent. Among the five potentially harmful *Gna11* variants generated, they selected the Asp195Gly variant for characterization, which effectively diminished intracellular calcium signaling *in vitro*, thereby validating it as a loss-of-function mutation. *In vivo*, both heterozygous and homozygous mutant mice mirrored human FHH2, exhibiting hypercalcemia with normal-to-elevated PTH levels and normal urinary calcium excretion. Notably, treating these mice with the calcimimetic cinacalcet successfully corrected their high plasma calcium and PTH concentrations (10).

In this report, we describe one Italian kindred whose proband referred to our outpatient clinic for a multinodular non-toxic goiter. During the diagnostic workup, mild asymptomatic hypercalcemia was detected, accompanied by normal PTH levels and a low calcium-creatinine clearance ratio (CCCR), suggesting a diagnosis of FHH. Genetic analysis confirmed the diagnosis, identifying a novel *GNA11* variant, p.His327LeufsTer150, consistent with FHH2.

## Case description

**Figure 1:**
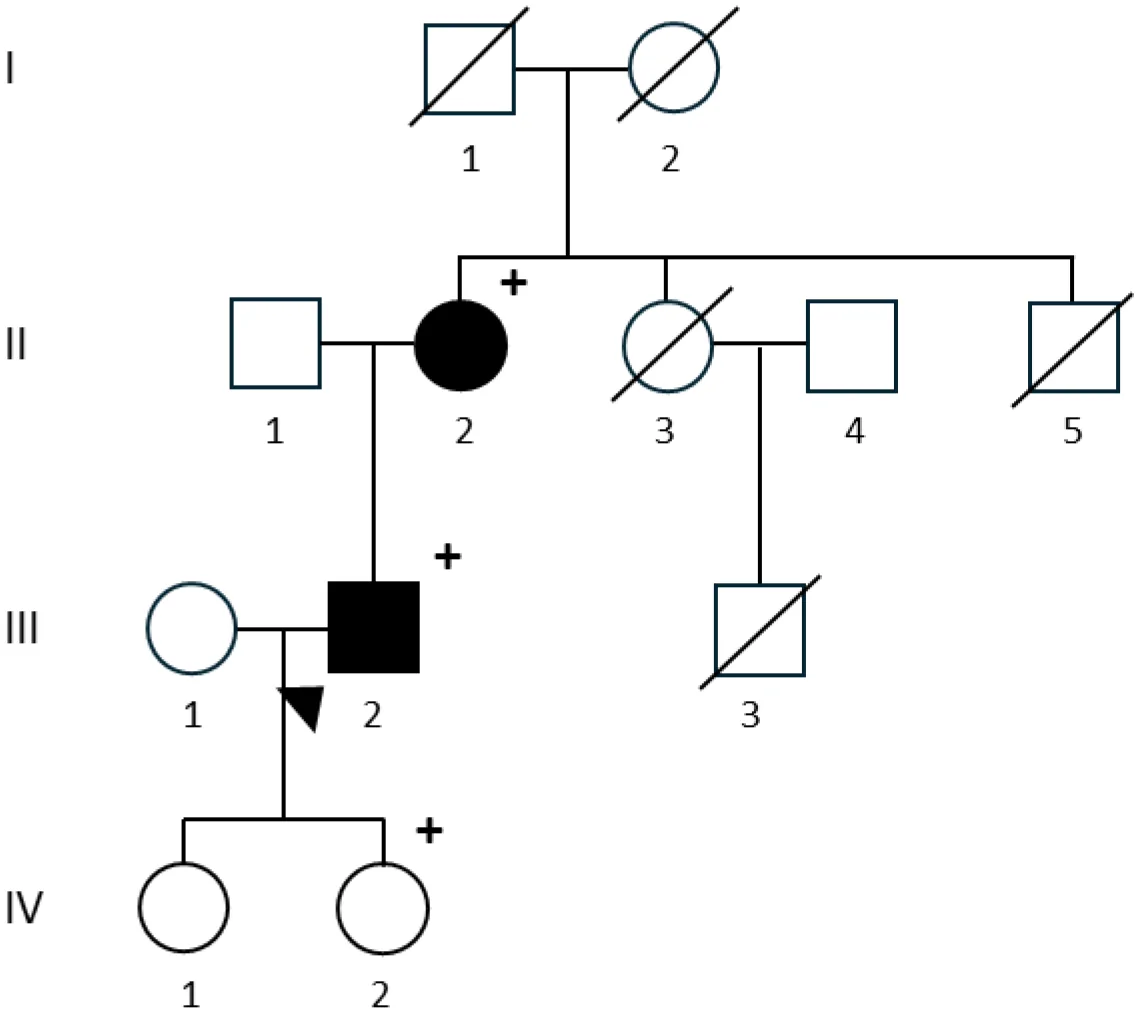
Genetic pedigree of the FHH2 family. Subjects are identified by generation and individual numbers and presented by a square (men) or a circle (women). Clinical status is indicated by the empty (unaffected) and filled symbols (affected). Slashed symbols indicate deceased individuals. The family’s proband is indicated by an arrow. The presence of the heterozygous deletion of two nucleotides c.980_981del (p.His327LeufsTer150) in the *GNA11* gene is indicated by a + in tested members.

In October 2020, a 39-year-old Caucasian male (III-2) was referred to our outpatient clinic for evaluation of a non-toxic multinodular goiter, which was incidentally discovered during a skull computed tomography scan (**Figure 1**). His medical history was notable for a traumatic skull fracture sustained in a car accident and the surgical removal of two lipomas from his back. No other significant findings emerged from his personal, physiological, or family medical history.

At the time of our evaluation, the patient was in good general condition. Physical examination revealed a mildly enlarged thyroid with a palpable 3-cm nodule at the lower pole of the left lobe, which was mobile on swallowing. No lymphadenopathies were detected in latero-cervical regions. Thyroid function tests were within normal limits, anti-thyroglobulin and anti-thyroperoxidase antibodies were negative, and serum calcitonin levels were undetectable. However, laboratory tests revealed an unexpected elevation in ionized serum calcium levels (1.42 mmol/L), while parathyroid hormone (PTH) and 25-hydroxyvitamin D (25[OH]D) levels were within normal ranges. Further biochemical investigations confirmed mild hypercalcemia and normal PTH levels (**Table 1**). Serum albumin, phosphate, and magnesium levels were within normal limits. Bone turnover markers and renal function tests were also normal. A 24-hour urinary calcium collection revealed inappropriately low-normal calcium excretion, with a low CCCR (**Table 1**). These findings were confirmed in a repeat 24-hour urinary collection. The patient reported no known family history of hypercalcemia.

**Table 1:** Summary of biochemical parameters of the proband at baseline and during follow-up.

Parameter	At diagnosis	6th month after total Tx	30th month after total Tx	Normal range
Serum total calcium (mg/dL)	10.3	9.6	10	8.6-10.2
Serum phosphate (mg/dL)	3.3	3.4	3.4	2.5-4.5
Serum magnesium (mg/dL)	2.1	2.1	2	1.7-2.5
Serum creatinine (mg/dL)	0.74	0.95	0.93	0.7-1.2
Ionized serum calcium (mmol/L)	1.44	1.32	1.28	1.13-1.32
Plasma PTH (ng/L)	28	29	30	8-40
25-OHD (ng/mL)	32	30	33	30-100
Serum BSAP (mcg/L)	15	–	–	5.7-32.9
Serum osteocalcin (mcg/L)	22.5	–	–	6.8-34
TSH (mUI/L)	0.643	4.2	3.7	0.4-4.0
fT3 (ng/L)	4.18	3.3	3.5	2.7-5.7
fT4 (ng/dL)	0.88	1.5	1.4	0.7-1.7
24-hour urinary calcium (mg)	135	75	–	100-300
24-hour urinary creatinine (mg)	1553	2000	–	1040-2350
CCCR	0.006	0.003	–	–

Tx, total thyroidectomy; 25-OHD, 25-hydroxyvitamin D; BSAP, bone specific alkaline phosphatase; CCCR, calcium–creatinine clearance ratio.

Neck ultrasound (US) confirmed the presence of a multinodular goiter, without any suspicious parathyroid lesions or latero-cervical lymphadenopathy. Cytological evaluation of the 3.5-cm nodule via fine-needle biopsy was consistent with a diagnosis of atypia of undetermined significance (Category III) according to the Bethesda system for Reporting Thyroid Cytopathology (11).

Abdominal ultrasound showed no evidence of kidney stones or nephrocalcinosis. Dual-energy X-ray absorptiometry (DXA) scan demonstrated normal bone mineral density at the hip, lumbar spine, and 1/3 distal radius.

Screening of serum calcium levels among the patient’s family members revealed mild hypercalcemia in his mother (II-2) and normal in both daughters (IV-1, IV-2) (**Figure 1**, **Table 2**). Further evaluation in the mother confirmed mild hypercalcemia, with PTH levels slightly elevated in the presence of vitamin D insufficiency (14 ng/mL). Low-normal urinary calcium excretion was evident, with a CCCR < 0.01. The patient started cholecalciferol treatment (2000 U.I./day), and six months later, ionized serum calcium was 1.35 mmol/L, PTH 38 ng/L, and CCCR = 0.01. The combination of the patient’s low urinary calcium excretion and the presence of hypercalcemia in the mother was suggestive of FHH. Consequently, after obtaining written informed consent, genetic testing was performed.

**Table 2:** Clinical, biochemical, and genetic findings of the proband’s relatives.

Family member	Age (years) at diagnosis/sex	Total Serum calcium (mg/dL)	Ionized Ca^2+^ (mmol/L)	PTH (ng/L)	24-hour urine calcium (mg/24 h)	CCCR	*GNA11* Genotype
Father (II-1)	61/M	9.7^a^	–	–	–	–	–
Mother (II-2)	60/F	10.3^b^	1.37^c^	41^d^	117	0.008	p.His327LeufsTer150
Daughter 1 (IV-1)	4/F	10.1^a^	–	26^d^	–	–	WT
Daughter 2 (IV-2)	15 months/F	10.5^a^	–	–	–	–	p.His327LeufsTer150
	6–11 months	10.6^a^	–	32.7^e^	–	–	

F, female; M, male; s-Ca2+, serum ionized Ca2+; CCCR, urinary calcium-to-creatinine clearance ratio. The Reference ranges are: a8.5-11 mg/dL; b8.6-10.2 mg/dL; c1.13-1.32 mmol/L; d8-40 ng/L; e2.5-73 ng/L.

For a non-toxic multinodular goiter with a suspected follicular lesion, the proband, in October 2022, underwent total thyroidectomy with a standard cervical approach. At neck exploration, no enlarged parathyroid glands were visualized by the surgeon. Histological examination confirmed a follicular adenoma in association with goiter. No parathyroid tissue was detected at histology. Six months after surgery and thereafter, the patient had normal serum calcium and PTH levels (**Table 1**).

**Figure 2:**
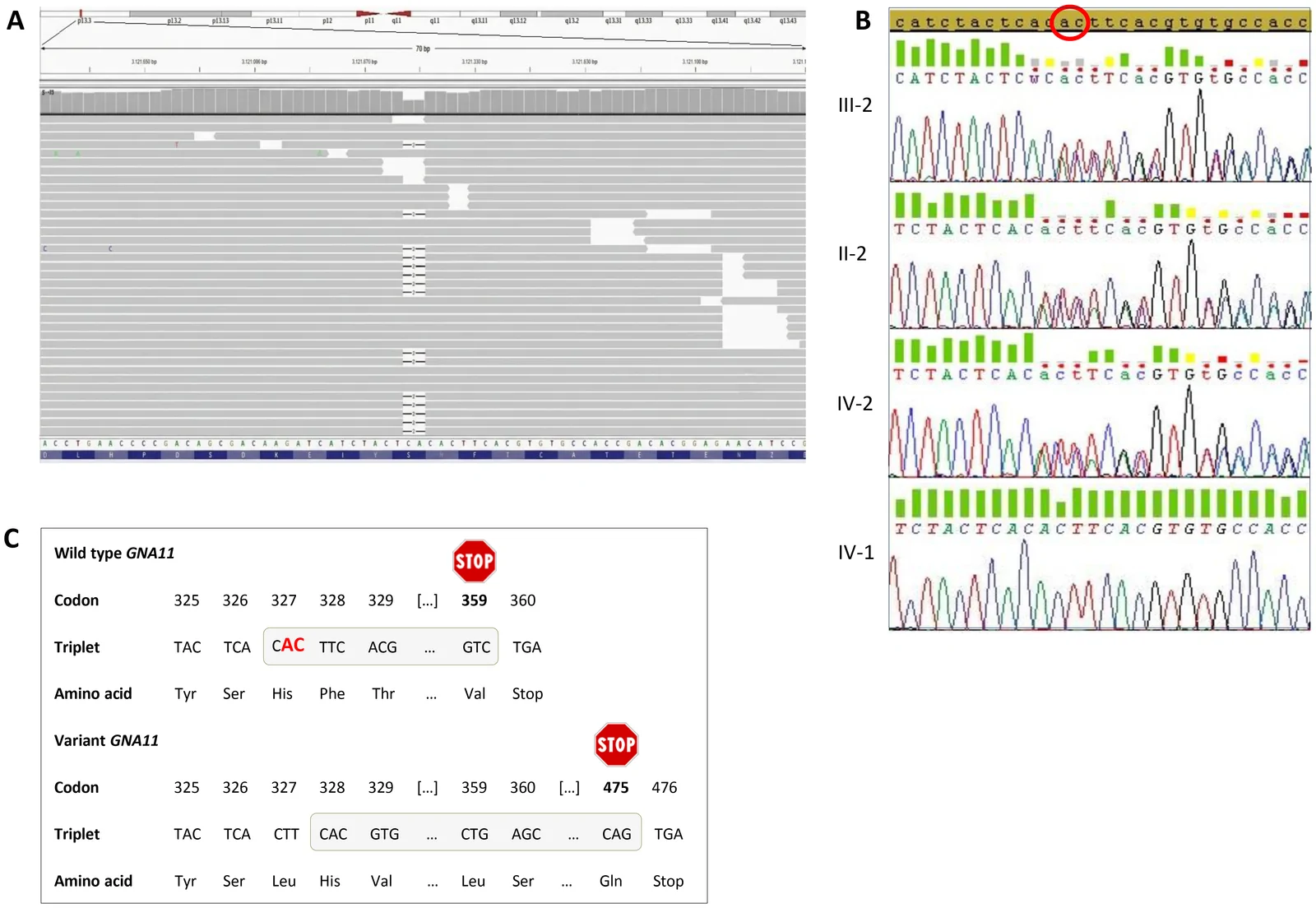
**(A)** Shows the Next Generation Sequencing (NGS) reads from the IGV (Integrative Genomic Viewer) software of a 70bp fragment of exon 7 of the GNA11 gene, showing the deletion of AC nucleotides (c.980_981del) in the proband III-2. The nucleotide and protein reference sequences are reported below (NM_002067.5). **(B)** confirms the NGS data by Sanger Sequencing, showing double peaks starting with the heterozygous deletion of nucleotides 980 and 981 (highlighted with a red circle in the nucleotide reference sequence reported above) in the proband (III-2), his mother (II-2), and youngest daughter (IV-2). The chromatogram sequence of the *GNA11* fragment from the oldest sister’s (IV-1) germline DNA is confirmed wild-type. **(C)** shows the predicted outcomes of the heterozygous 2-bp deletion of AC at nucleotides 980 and 981, as compared with the reference cDNA sequence. The 2-bp deletion, in red at codon 327, causes a shift in the reading frame downstream of the variant (p.His327Leufs*150), resulting in a protein that is 116 amino acids longer (ending with amino acid 475) than the original transcript (ending with amino acid 359).

Genetic testing identified a novel heterozygous germline variant in the *GNA11* gene: c.980_981del (p.His327LeufsTer150), located in exon 7 (**Figure 2**). The same variant was also detected in the patient’s mother (II-2) and youngest daughter (IV-2) (**Figure 2**). This frameshift deletion results in the formation of a new, elongated protein in which all codons downstream of the deletion are misread and a tail of 116 additional amino acids is introduced after residue 359, significantly altering the structure of the protein. Pathogenicity prediction tools like MutationTaster (https://www.mutationtaster.org/) and CADD v1.6 (https://cadd.gs.washington.edu/) predicted the variant to be deleterious with a score of 1.0, which reflects the highest confidence level for the prediction of pathogenicity, and a PHRED-scaled score of 34 (top 0.1% of deleterious alleles), respectively. The variant is absent from the population database (GnomAD) and lies within a strongly conserved stretch of amino acids. To our knowledge, this variant has never been previously reported and is classified as ‘likely pathogenic’ according to the American College of Medical Genetics and Genomics (ACMG) guidelines for the interpretation of sequence variants. This classification is supported by the combination of the following criteria: PVS1_strong (applied as a null variant in a gene where loss-of-function is an established disease mechanism, leading to an escaped nonsense-mediated mRNA decay that disrupts a critical functional domain), PM2_supporting (based on the complete absence of the variant from gnomAD genomes), PP1_supporting (based on co-segregation with phenotype in multiple affected family members in *GNA11* gene which is associated with Familial Hypocalciuric Hypercalcemia 2), and PP4_supporting (based on patient’s phenotype highly specific for FHH2, given the previous exclusion of mutations in the other FHH causative genes, namely *CASR* and *AP2S1*) (12–14).

## Clinical phenotype of the family members carrying the *GNA11* variant

The medical history of the 61-year-old proband’s mother (II-2) was unremarkable. She had no history of kidney stones or fractures. She was affected by non-toxic multinodular goiter, blood hypertension on treatment with β-blockers, and mild hypercholesterolemia. No history of hypercalcemia was known in her mother and father, who were deceased at the ages of 82 and 66 for type 2 diabetes-related complications and cardiac arrest, respectively.

The 15-month-old proband’s youngest daughter (IV-2) was born by full-term natural birth. Her motor and cognitive development has been normal. At her current age of 6 years and 11 months, she is in good health, with recent biochemical assessments confirming normal serum calcium and PTH levels (**Table 2**). A 24-hour urine collection for CCCR evaluation was not possible given the patient’s young age.

## Materials and methods

### Assays

Blood samples were obtained between 8 and 9 a.m. after an overnight fast. Serum calcium, creatinine, and phosphate were determined using standard methods. Plasma PTH was measured by a third-generation assay (DiaSorin LIAISON 1–84 PTH chemiluminescent immunoassay), and serum 25[OH]D was measured by a chemiluminescent immunoassay (IDS-iSYS); Bone-specific alkaline phosphatase (BSAP) by immunoenzymatic assay (OCTEIA Ostase BAP; IDS Ltd., Boldon, Tyne & Wear, UK), and serum N-MID osteocalcin (IDS Ltd., Boldon, UK) by ELISA. The reference ranges are reported in **Table 1**.

### Dual-energy X-ray absorptiometry (DXA)

Bone mineral density (BMD) was measured by DXA using Hologic QDR-4500 (Hologic Inc., Waltham, MA, USA) at the lumbar spine in posterior-anterior projection (L1–L4), femoral sites, total femur, and 1/3 distal radius. The coefficients of variation were 1.1% at lumbar spine, 1.2% at femoral neck, and 1.4% at 1/3 distal radius.

### DNA sequence analysis

Peripheral whole blood samples were collected, and genomic DNA was extracted with QIAsymphony automatic instrument), according to the manufacturer’s instruction (QIAGEN, Germany). We performed targeted sequencing covering all the coding exons and flanking regions of 3 genes known to be involved in FHH: *CASR* (NM_000388), *GNA11* (NM_002067), *AP2S1* (NM_004069). The Agilent online SureDesign tool was used to design the custom NGS panel. Captured and indexed library was sequenced on NextSeq 550Dx sequencer in 150 bp paired-end mode, according to the manufacturer’s protocol (Illumina, San Diego, CA, USA). Read alignment, variant calling, and annotation were performed with the Agilent SureCall software (Agilent Technologies, CA, USA). The coverage of each exon was analyzed in detail using IGV tool (Integrative Genomics Viewer, Broad Institute and the Regents of the University of California, USA). The obtained list of putative variants consisted of SNVs and small insertions and deletions relative to the reference genome (GRCh37/hg19). The identified variant in *GNA11* gene was validated by Sanger sequencing. DNA region containing the variant was amplified by polymerase chain reaction (PCR) using 5x Hot FirePol Blend Master Mix kit (Solis BioDyne, Estonia). The primers used for DNA amplification and for DNA sequencing (forward 5′-ACAAAGGGGCCCACGAGT-3′, reverse 5′- GTGTCTCCATCCCGTCTC-3′) were designed on the exon 7 sequence of the *GNA11* gene using the online Primer 3 software (PCR conditions available upon request). PCR product was purified using the Agencourt AMPure XP Kit (Beckman Coulter, CA, USA) and subjected to Sanger sequencing using Big Dye Terminator v3.1 Cycle sequencing kit (Applied Biosystems, CA, USA). The sequences obtained were purified through the Agencourt CLEANSEQ kit (Beckman Coulter) and sequenced with ABI Genetic Analyzer 3500 automatic sequencer (Applied Biosystems). Finally, the sequencing results were analyzed through SeqScape^®^ Software v3.1 (Applied Biosystems).

### *In silico* structural prediction

Three-dimensional (3D) structural modeling of both wild-type and mutated Gα11 proteins was performed using AlphaFold 3 (https://alphafoldserver.com/) (15). Model quality and topological reliability were assessed globally via the predicted Template Modeling score (pTM), to assess the accuracy of the global structural topology and locally via the predicted Local Distance Difference Test (pLDDT) score for each amino acid residue. The server also computed the global fraction of structurally disordered residues to identify intrinsically disordered regions (IDRs).

## Discussion

FHH is a typically benign, non-progressive disorder that affects CaSR signal transduction. The estimated prevalence of FHH was first reported by Hinnie et al., who reported a prevalence of approximately 1 in 78,000 in a combined Scottish population of 1,473,700 residents in Greater Glasgow and Lanarkshire Health Boards (16). More recently, Dershem et al. reported a prevalence of FHH1 of 74.1 per 100,000 in a cohort of 51,289 individuals (DiscovEHR cohort) predominantly of northern European descent (17). The three subtypes of FHH present a similar phenotype, characterized by lifelong asymptomatic mild hypercalcemia, normal or slightly elevated PTH levels, and relative hypocalciuria. However, some studies have described a more symptomatic phenotype in FHH3, characterized by more severe hypercalcemia, hypermagnesemia, and pronounced hypocalciuria (18). Additionally, low BMD and cognitive impairment have been observed in FHH3 patients (18–20). FHH is generally considered a benign condition, and most patients do not require specific medical or surgical treatment, only periodic monitoring over time. However, in some cases of symptomatic moderate-to-severe hypercalcemia, complicated by acute conditions such as chronic pancreatitis, chondrocalcinosis, and nephrolithiasis, the calcimimetic cinacalcet - an allosteric modulator of CaSR that amplifies its sensitivity - has been used to reduce hypercalcemia (6, 21–26).

FHH2 is the rarest form of FHH, caused by inactivating mutations in the *GNA11* gene, which is located on chromosome 19p13.3 and encodes the G protein alpha subunit (Gα11) (7). Gα11, composed of a GTPase domain, binds and hydrolyzes guanosine triphosphate (GTP), transducing signals from CaSR through cytoplasmic inositol 1,4,5-triphosphate and the MAPK cascade, and a helical domain that shields the nucleotide in a core (27).

**Figure 3:**
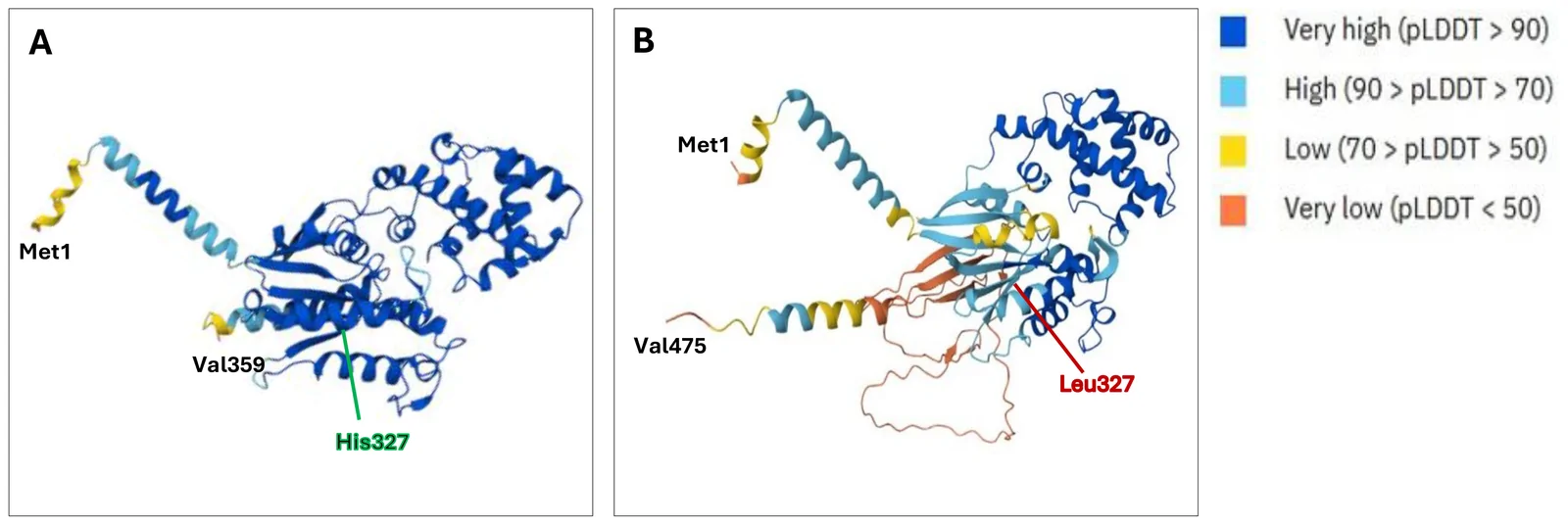
Structural prediction of the wild-type **(A)** and variant **(B)** Gα11 proteins according to AlphaFold3 (https://alphafoldserver.com). The per-residue confidence scores (pLDDT, predicted Local Distance Difference Test) evaluate the local reliability of the predicted atom distances on a scale from 0 to 100. High values (pLDDT > 70, shown in blue and cyan) indicate highly confident, rigid structural domains, whereas low values (pLDDT < 50, in yellow/orange) correspond to regions with high conformational flexibility. The existence of a low-confidence C-terminal tail in the variant model, along with a calculated global fraction of disordered residues of 0.29, indicates the absence of a defined globular fold and the presence of a massive intrinsically disordered region (IDR) spanning the last 148 amino acids. The predicted Template Modeling (pTM) score that evaluates the accuracy of the global structural topology ranges from 0 to 1, where values greater than 0.5 indicate that the model shares the same global fold as the native protein, and scores exceeding 0.7 signify a highly reliable backbone topology. The calculated pTM score of the variant Gα11 model was 0.66, confirming that the introduction of the 148-amino-acid aberrant tail does not destabilize or disrupt the core globular architecture of the receptor-coupling domains. The first, the last, and the residues at the site of the 2-base-pair deletion are indicated for both the wild-type **(A)** and variant **(B)** proteins.

In the present study, we report a novel heterozygous variant in exon 7 of the *GNA11* gene, c.980_981del (p.His327LeufsTer150), identified in a patient with FHH. The His327 residue, the first amino acid affected by the deletion, is located in the β6 strand of the GTPase domain, which plays a key role in guanine nucleotide binding (**Figure 3**). Notably, the deletion of the two nucleotides results in a frameshift downstream of the variant, leading to the production of a protein that is 116 amino acid residues longer than the original transcript. The addition of a non-native C-terminal “tail,” resulting from the incorporation of a portion of the 3’ UTR into the coding sequence, likely disrupts proper tertiary folding, rendering the protein unstable or non-functional. Our *in silico* structural analysis using AlphaFold3 supports a further rationale for the pathogenicity of the identified variant. The model indicates that the core globular fold of the Gα11 subunit in the variant protein remains structurally intact, whereas the frameshift deletion introduces an intrinsically disordered region (IDR) at the 148-amino-acid C-terminal extension, disrupting the native α5 helix. Because this domain is crucial for coupling with G-protein-coupled receptors (GPCRs), the high conformational flexibility and steric hindrance of this aberrant tail are likely to impair receptor-effector interactions, leading to a loss-of-function in downstream signaling. Notably, this variant is expected to escape nonsense-mediated mRNA decay (NMD) because the frameshift deletion is located within the final exon (exon 7). Since NMD is typically triggered only when a premature termination codon is positioned upstream of exon junction complexes (EJCs)—which are deposited at splice sites during mRNA processing—this dysfunctional transcript evades degradation (28). Consequently, the mRNA is available for translation into a protein with an aberrant, elongated C-terminus. The evasion of the variant transcript from the mRNA control machinery shifts the pathogenic mechanism from the simple lack of protein production to a more complex model where the fate of the non-degraded transcript depends on post-translational quality control. On one hand, the maintenance in the variant Gα11 model of a reliable core global architecture, as suggested by our AlphaFold3 analysis, potentially allows the aberrant protein to persist within the cell and remain relatively stable. In this scenario, the variant protein could exert a dominant-negative effect or induce steric hindrance at the plasma membrane, locking or blocking the access of wild-type partners to GPCRs and disrupting downstream intracellular cascades. On the other hand, the structural prediction reveals that the frameshift replaces the native α5 helix with a 148-amino-acid intrinsically disordered region (IDR), disrupting the core GTPase domain topology (**Figure 3**). Functionally, this domain must alternate between open and closed conformations to exchange GDP for GTP; however, in the variant model, the flexible C-terminal IDR might fold over the binding pocket, physically blocking nucleotide exchange. Consequently, the variant G-protein may become fixed in a perpetually “empty” open conformation, marking it for post-translational clearance by different cellular quality control pathways. During synthesis, it can be intercepted within the endoplasmic reticulum by the ER-associated degradation (ERAD) machinery, preventing its trafficking to the cell surface. Alternatively, if the altered protein reaches the cytoplasm or plasma membrane, the abnormal exposure of hydrophobic residues of the IDR should create thermodynamic instability that targets the protein for rapid destruction via the general ubiquitin-proteasome system (UPS). Therefore, while functional assays are required to definitively determine whether the variant acts through a dominant-negative mechanism or via rapid proteasomal clearance, both potential loss-of-function models provide a theoretical rationale for how this genetic alteration might impair protein function.

Although the *GNA11* frameshift variant segregates with the classic FHH biochemical phenotype in the proband (III-2) and his mother (II-2), the absence of a hypercalcemic phenotype in the proband’s daughter carrying the variant (IV-2) could be misinterpreted as a “non-segregation” event that would preclude the application of the ACMG PP1 criterion. According to the recent ClinGen Sequence Variant Interpretation recommendations, implemented in the updated ACMG v4.0 framework, the co-segregation analysis allows for the conservative exclusion of young, unaffected carriers in families showing age-dependent penetrance or variable expressivity (12). These updated guidelines overcome the traditional requirement of at least three informative meioses and justify applying the PP1 supporting evidence based on the remaining informative meiosis, thus reinforcing the pathogenic classification of this variant. Unfortunately, from a clinical perspective, ionized calcium, which is required to assess calcium status accurately, was not available for subject IV-2. Notably, in patients with mild hypercalcemia, measurements of total calcium alone would miss 45% of hypercalcemic subjects (29). Moreover, CCCR was also not available. It may also be hypothesized that, during childhood, intense skeletal mineralization sequesters calcium into bone, temporarily masking downstream *GNA11* defects within normal pediatric ranges. Post-puberty, as skeletal growth slows, this protective effect is expected to diminish, manifesting the biological ‘set-point’ shift that brings to full biochemical expressivity. In agreement with our data, Boisen et al. reported a 32-year-old FHH2-affected relative who was normocalcemic at the time of observation, suggesting a variable penetrance of the disease, as already reported for FHH1 (17, 30). Regular monitoring of serum calcium, PTH, and urinary calcium excretion during adolescence is crucial to observe the expected phenotypic changes in the proband’s daughter carrying the variant.

While, as stated above, functional studies are still needed to confirm its effect, the variant is predicted to produce a stable but dysfunctional extended protein. This hypothesis is supported by *in silico* predictions indicating a deleterious effect, alongside the ACMG-based classification that supports the clinical interpretation of the variant as likely pathogenic.

To date, only a few studies have described *GNA11* mutations associated with FHH2 in unrelated patients from different families (4–9). Six of these are missense variants, and one is an in-frame deletion of isoleucine at residue 200 (**Table 3**). The three-dimensional modeling of the protein, derived from the crystal structure of Gαq, which shares 90% amino acid identity with Gα11, shows that all variants affect the GTPase domain, impairing CaSR-Gα11 coupling and PLC activation, or alter the helical domain, influencing downstream signaling mediated by the Gα11 protein upon CaSR activation. *In vitro* studies using HEK293 cells stably expressing CaSR demonstrated the loss-of-function characteristics of five of these variants, with a variable grade of disturbances of the CaSR signal transduction (**Table 3**) (4). Conversely, functional studies of the nonsynonymous p.Met87Val variant, located in the helical domain away from the GDP binding site and classified as a variant of uncertain significance, revealed no differences in intracellular calcium responses to different extracellular calcium concentrations, suggesting the benign nature of the variant (7). Koca et al. first reported a homozygous missense c.301T>C (p.Tyr101His) variant of *GNA11* in a 14-year-old male presenting with mild hypercalcemia (**Table 3**). The same variant was detected in the heterozygous state in both parents. Although both parents had normal serum calcium levels, their spot urinary fractional calcium excretion was low (9).

**Table 3:** Summary of *GNA11* variants identified in FHH2 reported in the literature and phenotypical characteristics of FHH2 probands.

Reference	Clinical findings		Biochemical findings		Nucleotidic *GNA11* variant/het,hom	Protein Gα11 variant	*In vitro* studies			*In silico* analysis
	Age at presentation or diagnosis (yr)/Sex	Family history (affected members)	Serum calcium mmol/L (n.r.)	PTH pmol/L (n.r.)			EC_50_^a^ (95% CI)	*P* value (vs. non-mutant plasmid)	Functional consequences of the variant	
Nesbit 2013 et al. (4)	45/F	Yes (10)	2.70 (2.10-2.50)	2.70 (<5)	c.598_600del/het	p.Ile200del	2.57 (2.53-2.61)	<0.001	Disease causing	NA
Nesbit 2013 et al. (4)	54/M	NA	2.63 (2.10-2.50)	5.0 (1.3-7.6)	c.404T>A/het	p.Leu135Gln	3.12 (3.05-3.19)	<0.001	Disease causing	NA
Gorvin 2016 et al. (5)	65/F	No	2.77 (2.20-2.60)	5.9 (1.0-7.0)	c.161C>T/het	p.Thr54Met	3.88 (3.76-4.01)	<0.01	Disease causing	SIFT: damaging MutationTaster: likely disease-causing
Gorvin 2018 et al. (6)	33/M	Yes (5)	_i_Ca^++^ 1.42 (1.16-1.30)	10.3 (0.8-7.7)	c.659T>C/het	p.Phe220Ser	2.93 (2.80-3.07)	<0.001	Disease causing	Polyphen2: damaging MutationTaster: likely disease-causing
Howles 2023 et al. (7)	19/F	NA	2.74 (2.10-2.60)	4.69 (1.3-7.6)	c.259A>G/het	p.Met87Val	3.15 (3.02-3.28)	NS	No effect	Alamut: VUS SIFT: Tolerated MutationTaster: Benign MetaLR: damaging
Boisen 2024 et al. (8)	75/F	Yes (5)	2.68 (2.15-2.51)	4.38 (1.6-6.9)	c.1039A>G/het	p.Thr347Ala	2.36 (± 0.01)	0.038	Disease causing	NA
Koca 2025 et al. (9)	14/M	Yes (3^b^)	2.89 (2.15-2.55)	7.95 (1.59-6.89)	c.301T >C/hom	p.Tyr101His	NA	–	–	SIFT: damaging PolyPhen: damaging ACMG: VUS
Present study	39/M	Yes	2.57 (2.15-2.55)	2.97 (0.85-4.24)	c.980_981del/het	p.His327LeufsTer150	NA	–	–	MutationTaster: deleterious CADD: deleterious

yr, years; F, female; M, male; iCa^++^, ionized calcium; PTH, parathyroid hormone; nr, normal range; het, heterozygous; hom, homozygous; CI, confidence interval; NA, not available; NS, not significant; VUS, variant of unknown significance.

^a^ The extracellular calcium concentration that produces a half-maximal (EC_50_) increase in intracellular calcium, primarily mediated by the Calcium-Sensing Receptor (CaSR).

^b^ The proband’s parents were heterozygous for the variant but biochemically normal.

Like the family described in our study (**Table 3**), all reported patients exhibited mild hypercalcemia with normal serum PTH levels and low urinary CCCR (<0.01). The only exception was the patient carrying the p.Phe220Ser variant, who presented with a more severe phenotype. This included hypercalcemia, elevated serum PTH levels, hypophosphatemia, and persistently low urinary CCCR (**Table 3**). The clinical picture was further complicated by hypercalcemia-related symptoms such as headaches, constipation, and pruritus, ultimately necessitating treatment with cinacalcet (6).

The patient described herein underwent total thyroidectomy for a follicular lesion of undetermined significance. Following surgery, repeated measurements of serum calcium were unexpectedly within the normal range. Notably, before surgery, at least six measurements of total serum calcium were at the upper limit of normal or mildly elevated (ranging from 10.2 to 10.3 mg/dL), while ionized serum calcium levels were consistently elevated (ranging from 1.38 to 1.42 mmol/L). FHH2 is known to exhibit variable penetrance and biochemical expressivity; therefore, normalization of serum calcium after thyroidectomy may reflect spontaneous biochemical fluctuation of the FHH2 phenotype rather than a direct effect of surgery. An alternative explanation could be a subtle parathyroid impairment related to surgical manipulation or vascular compromise. Even a mild and persistent reduction in PTH secretion, while remaining within the reference range, might be sufficient to normalize serum calcium levels in a patient with altered calcium-sensing signaling. Post-operative parathyroid impairment should be regarded as a speculative and indirect explanation for the observed biochemical changes rather than a demonstrated mechanism in our patient. Notably, in the patient, post-operative PTH concentrations were comparable to pre-surgical values, arguing against a clinically relevant reduction in parathyroid function. The preserved PTH levels and the absence of parathyroid tissue on histological examination may argue against significant parathyroid injury, and that alternative mechanisms may have contributed to the post-operative biochemical findings. regarding the potential contribution of post-thyroidectomy changes in thyroid status. Indeed, TSH levels slightly increased following surgery (data not shown), and experimental and clinical evidence suggests that TSH may have direct inhibitory effects on osteoclast differentiation and activity, thereby reducing bone resorption and calcium release from the skeleton (31). Therefore, it is conceivable that the post-operative increase in TSH contributed, at least in part, to the normalization of serum calcium levels observed during follow-up. However, TSH levels rapidly returned to the normal range after surgery, making it unlikely that this mechanism alone could fully account for the sustained normalization of serum calcium. Nevertheless, a transient effect of post-operative changes in thyroid status on bone turnover and calcium homeostasis cannot be excluded. Thus, considering all previous considerations, the cause of the sustained normalization of serum calcium remains uncertain, and definitive evidence of a causal relationship cannot be drawn.

The youngest proband’s daughter (IV-2) had normal total calcium based on two measurements; unfortunately, ionized calcium, which is required to accurately assess calcium status, was not available. Notably, in patients with mild hypercalcemia, measurements of total calcium alone would miss 45% of hypercalcemic subjects (29). Moreover, CCCR was also not available. In agreement with our data, Boisen et al. reported a 32-year-old FHH2 affected relative who was normocalcemic at the time of observation, suggesting a variable penetrance of the disease, as already reported for FHH1 (17, 30).

In conclusion, FHH is a benign condition, typically presenting with mild, non-progressive hypercalcemia and without an increased risk of renal or skeletal complications. This case highlights the biochemical variability of FHH2 and suggests that normalization of serum calcium, as documented during follow-up, does not necessarily exclude the diagnosis. It also underscores the importance of integrating genetic findings with longitudinal biochemical data.

## Data Availability

The raw data supporting the conclusions of this article will be made available by the authors, without undue reservation. The novel germline GNA11 variant has been submitted to the NCBI ClinVar database with accession number: SCV007653944.1.
